# Non-linear relationship between high-density lipoprotein cholesterol and incident diabetes mellitus: a secondary retrospective analysis based on a Japanese cohort study

**DOI:** 10.1186/s12902-022-01074-8

**Published:** 2022-06-18

**Authors:** Changchun Cao, Haofei Hu, Xiaodan Zheng, Xiaohua Zhang, Yulong Wang, Yongcheng He

**Affiliations:** 1 Department of Rehabilitation, Shenzhen Dapeng New District Nan’ao People’s Hospital, No. 6 Renmin Road, Dapeng New Distric, Shenzhen, 518000 Guangdong Province China; 2 Department of Nephrology, The First Affiliated Hospital of Shenzhen University, Shenzhen Second People’s Hospital, Shenzhen, 518035 Guangdong China; 3 Department of Nephrology, Shenzhen Hengsheng Hospital, No. 20 Yintian Road, Xixiang Street, Baoan District, Shenzhen, 518000 Guangdong Province China

**Keywords:** High-density lipoprotein cholesterol, Incident diabetes mellitus, Nonlinearity

## Abstract

**Background and objective:**

High-density lipoprotein cholesterol (HDL-C) may be directly involved in glucose metabolism by enhancing insulin sensitivity and insulin secretion. This current study aimed to explore the association between HDL-C and the risk of diabetes mellitus (DM) in Japanese population.

**Methods:**

This retrospective cohort study was based on a publicly available DRYAD dataset. We enrolled 15,388 Japanese participants who received medical examinations from 2004 to 2015 at Murakami Memorial Hospital. Our study selected HDL-C at baseline and incident DM during follow-up as the target independent variable and the dependent variable, respectively. Cox proportional-hazards regression was used to investigate the association between HDL-C and DM, generalized additive models to identify non-linear relationships.

**Results:**

After adjusting for the demographic and clinical covariates, the result showed low HDL-C levels were associated with increased risk for diabetes (HR = 0.54, 95%CI (0.35, 0.82)). The results remained robust in a series of sensitive analysis. A non-linear relationship was detected between HDL-C and incident DM with an inflection point of HDL-C at 1.72 mmol/L (Log-likelihood ratio test *P* = 0.005). Subgroup analysis showed that a stronger association could be found in ex-smokers and current-smokers. The same trend was also seen in the community with hypertension (*P* for interaction = 0.010, HR = 1.324).

**Conclusion:**

This study demonstrates a negative and non-linear relationship between HDL-C and diabetes in the Japanese population. There is a threshold effect between HDL-C and diabetes. When HDL-C is lower than 1.72 mmol/L, the decreased HDL-C levels were associated with an increased risk for diabetes.

**Supplementary Information:**

The online version contains supplementary material available at 10.1186/s12902-022-01074-8.

## Introduction

Diabetes has become one of the most critical public health problems in the world. The International Diabetes Federation reported that the global prevalence of age-standardized diabetes, estimated at 9.3% in 2019, will rise to 10.2% by 2030 and 10.9% by 2045 [1]. As one of the most common chronic diseases, diabetes has brought a substantial economic burden to patients and their countries [2]. Diabetes is a metabolic disease and often accompanied by dyslipidemia, which is characterized by a decrease in serum high-density lipoprotein cholesterol (HDL-C) and increased triglycerides (TG) [3–6]. HDL-C had been recognized as a cardiovascular protective factor by most scholars [7]. Some studies had also revealed that lower level of HDL-C was related to a higher risk of DM [8, 9]. However, a recent multicenter retrospective study involving 114,787 participants demonstrated that high levels of HDL-C increased the risk of DM [10]. Some clinical trials also have shown that some drugs that increase HDL-C levels, such as cholesteryl ester transfer protein (CETP) inhibitors and niacin, cannot decrease the risk of cardiovascular and cerebrovascular diseases [11–13]. The current research on the relationship between HDL-C and diabetes risk is still controversial, and it is unclear whether the risk of diabetes is related to HDL-C levels. Therefore, this current study aimed to explore the association between HDL-C levels and the risk of incident DM in the population through a public database from Japan.

Our study conducted a secondary analysis according to a previous public database from a longitudinal study that showed ectopic obesity increased the risk of diabetes.

## Research objects and methods

### Data source

This study conducted a secondary analysis from the DRYAD database(www.Datadryad.org). We have free access to the raw data from this site and analyzed the data. (Dryad data package: Okamura T, Hashimoto Y, Hamaguchi M, Obora A, Kojima T, Fukui M (2019) Data from Ectopic fat obesity presents the greatest risk for incident diabetes: a population-based longitudinal study. Dryad Digital Repository. https://datadryad.org/stash/dataset/doi:10.5061%2Fdryad.8q0p192) [14]. Variables contained in the public database were as follows: age, gender, body mass index (BMI), waist circumference (WC), ethanol consumption, smoking status, exercise habit, fatty liver, and baseline levels of systolic blood pressure (SBP), diastolic blood pressure (DBP), alanine aminotransferase (ALT), aspartate aminotransferase (AST), total cholesterol (TC), TG, HDL-C, fasting blood glucose (FPG), glycosylated hemoglobin (HbA1c), day of follow-up and incident diabetes at follow up. The authors of the original research waive all copyrights to these data. Therefore, we could conduct a secondary analysis by using these databases without compromising the authors’ rights. All methods were performed in accordance with the relevant guidelines and regulations by including a statement in the Declarations section.

### Study participants

We obtained data from a database which was provided by the Murakami Memorial Hospital in Japan. This database contained 20,944 participants who received medical examinations from 2004 to 2015. The exclusion criteria of the previous original research were as follows**:** (1) alcoholic fatty liver disease, (2) viral hepatitis (detection of hepatitis B antigen and hepatitis C antibody at baseline), (3) using any medication at baseline, (4) diabetes at baseline, (5) missing data of covariates, (6) FPG ≥ 6.1 mmol/L. Okamura and his colleagues selected 15,464 participants finally. The original retrospective cohort study described inclusion/exclusion criteria and trial outcome measures [14]. The ethics committee approved the original research of Murakami Memorial Hospital and informed consent was obtained from all participants. For further analysis, 126 participants with outliers of HDL-C, including HDL-C less than the mean minus three standard deviations (SD) or greater than the mean plus three SD, were excluded [15]. Eventually, 15,338 subjects (8397 male and 6941 female) were included in our study’s data analysis.

### Study design and measurement of variables

The previous original study obtained medical history and lifestyle factors of all participants through a standardized self-management questionnaire. The trained staff conducted the clinical measurements, including body weight, waist circumference height, and blood pressure. Laboratory inspection results are collected under standardized conditions and conducted in accordance with uniform procedures. The previous original study defined regular exercise as playing any type of exercise > 1 × /week [16]. The previous original study assessed ethanol consumption by asking the participants about their alcohol consumption in the previous month, then estimating the mean ethanol intake per week [14]. The previous original study defined visceral fat obesity as a waist circumference ≥ 90 cm in men or ≥ 80 cm in women [17]. The target independent variable is HDL-C obtained at baseline. The dependent variable is incident diabetes obtained in the follow up. As a retrospective cohort study, our study reduced the possibility of selection bias and observation bias.

Diagnosis of incident diabetes.

DM was defined as HbA1c not lower than 6.5%, FPG not lower than 7 mmol/l [18] or self-reported during the follow-up period.

### Statistical analysis

We used EmpowerStats software (www.empowerstats.com, X&Y solutions, Inc., Boston, MA, USA) and R (http://www.R-project.org) for data analysis.

Participants were divided into four groups according to the quartile of HDL-C. Continuous variables were presented as mean ± standard deviation when the data obeyed normal distribution and expressed as the median or interquartile range when the data obeyed skewed distribution. We used frequencies and percentages to present categorical variables. We also analyzed differences among groups by performing the Kruskal–Wallis H test, the one-way ANOVA and the chi-square test. Univariate and multivariate Cox regression analysis were applied to evaluate the association between HDL-C and diabetes risk. The Cox regression model was used to calculate the hazard ratio (HR) and 95% confidence interval (95% CI) of diabetes caused by HDL-C. According to the STROBE statement’s recommendation [19], we used three models to describe HDL-C and DM’s relationship. The crude model included only HDL-C as the independent variable. We adjusted for gender, age, smoking, ethanol consumption, regular exerciser, SBP, DBP, BMI and WC in the model I. Model II included all the variables in the model I and further adjusted for TC, TG, HbA1c, FPG. The confounding covariates with original effect size (hazard ratio) change > 10% or *P*-value of regression coefficient < 0.1 can be added to the model as confounding factors [20]. In order to ensure the robustness of the result, we conducted a series of sensitivity analysis. We transformed HDL-C into categorical variables based on quartiles and calculated the *P*-value for trend. The aim was to evaluate a potential non-linear relationship of HDL-C with the risk of DM. We performed a weighted generalized additive model (GAM) model to adjust for the covariates in Mode III, because the generalized linear model has limitations in dealing with nonlinearities. Additionally, we explored the potential for unmeasured confounding between HDL-C and DM risk by calculating E-values [21].

We used a GAM to evaluate the non-linear relationship between HDL-C levels and the incidence of diabetes, and further used a two-piece linear regression model to find the inflection point. The most appropriate model for describing the relationship between HDL-C and DM risk was determined by using log likelihood ratio test. The Cox proportional hazard model was used to conduct subgroup (age, gender, ethanol consumption, smoking status, regular exerciser, SBP, DBP, BMI, WC, fatty liver) analysis to further verify the robustness of the results. In addition, a likelihood ratio test was performed to evaluate the interaction between subgroups. We used the Kaplan–Meier method by using the time-to-first event for each endpoint to compare survival estimates and cumulative event rates. Kaplan–Meier HRs for adverse events and their corresponding 95% CIs were compared by the log-rank test. A two-sided *P* < 0.05 was considered significant.

## Results

Our study included 15,388 participants, of whom 54.7% were men and 45.3% were women. The mean ± SD age of the participants was 43.7 ± 8.9 years. After a maximum of 4732 days of follow-up (median 1967 days), 373 people finally developed diabetes. The mean ± SD HDL-C was 1.5 ± 0.4 mmol/L. The mean ± SD FPG, BMI, and WC were 5.2 ± 0.4 mmol/L, 22.1 ± 3.1 kg/m^2^, and 76.5 ± 9.1 cm.

### Baseline demographic and clinical characteristics

Table 1 shows the baseline demographic and clinical characteristics of participants. Participants were divided into subgroups according to HDL-C quartiles (≤ 1.164, 1.164–1.407, 1.407–1.696, > 1.696). In the lowest HDL-C group, participants commonly had higher age, BMI, WC, SBP, DBP, FPG, HbA1c, TG, ethanol consumption, higher proportion of men, fatty liver, and current smokers. In the highest HDL-C group, we found that participants generally had higher TC and they were regular exerciser.

**Table 1:** The Baseline Characteristics of participants

HDL-C(mmol/L)	Q1(≤ 1.16)	Q2(1.16 to ≤ 1.41)	Q3(1.41 to ≤ 1.70)	Q4(> 1.70)	*P*-value
Participants	3752	3895	3845	3846	
Gender					< 0.001
Women	568 (15.14%)	1419 (36.43%)^a^	2122 (55.19%)^ab^	2832 (73.63%)^abc^	
Men	3184 (84.86%)	2476 (63.57%)	1723 (44.81%)	1014 (26.37%)	
Age(years)	44.49 ± 9.03	43.63 ± 8.96^a^	43.31 ± 8.77^a^	43.36 ± 8.78^a^	< 0.001
Ethanol consumption(g/week)	4.20(0, 66.00)	1.00(0, 72.00)	1.00(0, 60.00)	1.00(0, 54.00)^abc^	< 0.001
Smoking status					< 0.001
Never-smoker	1436 (38.27%)	2114 (54.27%)^a^	2484 (64.60%)^ab^	2906 (75.56%)^abc^	
Ex-smoker	863 (23.00%)	821 (21.08%)	694 (18.05%)	550 (14.30%)	
Current-smoker	1453 (38.73%)	960 (24.65%)	667 (17.35%)	390 (10.14%)	
Regular exerciser					< 0.001
No	3190 (85.02%)	3226 (82.82%)^a^	3156 (82.08%)^a^	3085 (80.21%)^abc^	
Yes	562 (14.98%)	669 (17.18%)	689 (17.92%)	761 (19.79%)	
SBP (mmHg)	118.88 ± 14.75	115.96 ± 14.97^a^	112.67 ± 14.53^ab^	110.63 ± 14.31^abc^	< 0.001
DBP (mmHg)	75.00 ± 10.21	72.62 ± 10.41^a^	70.29 ± 10.23^ab^	68.55 ± 10.03^abc^	< 0.001
BMI (kg/m^2^)	23.95 ± 3.11	22.61 ± 3.08^a^	21.50 ± 2.71^ab^	20.52 ± 2.47^abc^	< 0.001
WC (cm)	82.37 ± 8.30	78.17 ± 8.52^a^	74.47 ± 8.13^ab^	71.20 ± 7.40^abc^	< 0.001
Fatty liver					< 0.001
No	2339 (62.34%)	3081 (79.10%)^a^	3491 (90.79%)^ab^	3693 (96.02%)^abc^	
Yes	1413 (37.66%)	814 (20.90%)	354 (9.21%)	153 (3.98%)	
ALT (IU/L)	21(16.00,30.00)	17 (13.00,24.00)^a^	15 (12.00,20.00)^ab^	14(11.00,19.00)^abc^	< 0.001
AST (IU/L)	18 (15.00,22.00)	17 (14.00,21.00)^a^	17 (14.00,20.00)^ab^	17 (13.00,20.00)^abc^	< 0.001
HDL-C (mmol/L)	1.00 ± 0.12	1.29 ± 0.07^a^	1.54 ± 0.08^ab^	1.97 ± 0.22^abc^	< 0.001
TG (mmol/L)	1.20 (0.83,1.73)	0.82 (0.56,1.17)^a^	0.64 (0.45,0.88)^ab^	0.53 (0.38,0.72)^abc^	< 0.001
TC (mmol/L)	5.07 ± 0.89	5.07 ± 0.89	5.07 ± 0.84	5.27 ± 0.80^abc^	< 0.001
HbA1c (%)	5.19 ± 0.34	5.18 ± 0.33	5.15 ± 0.31^ab^	5.17 ± 0.30^ac^	< 0.001
FPG (mmol/L)	5.30 ± 0.38	5.20 ± 0.40^a^	5.11 ± 0.41^ab^	5.04 ± 0.41^abc^	< 0.001

Values are n (%) or mean ± SD

*SBP* Systolic blood pressures, *DBP* Diastolic blood pressures, *BMI* Body mass index, *WC* Waist circumference, *ALT* Alanine aminotransferase, *AST* Aspartate aminotransferase, *HDL-C* High-density lipoprotein cholesterol, *TC* Total cholesterol, *TG* Triglycerides, *HbA1c* Hemoglobin A1c, *FPG* Fasting plasma glucose

^a^represents other groups (Q2, Q3, Q4) compared with Q1, *P* < 0.05

^b^represents other groups (Q3, Q4) compared with Q2, *P* < 0.05

^c^represents other groups (Q4) compared with Q3, *P* < 0.05

### Univariate analysis

**Fig. 1:**
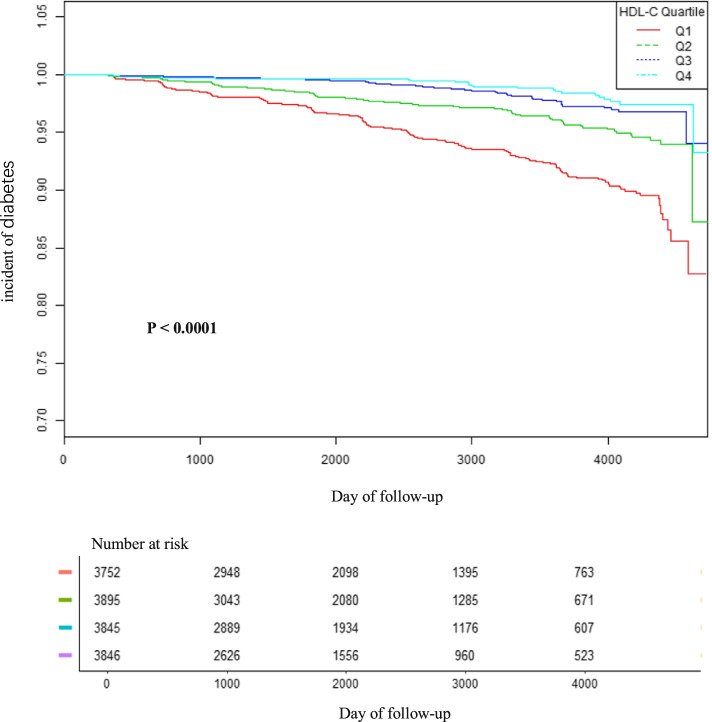
Kaplan–Meier event-free survival curve. Kaplan–Meier event-free survival curve. Kaplan–Meier analysis of incident diabetes based on HDL-C quartiles (log-rank, *P* < 0.0001)

Table 2 showed the univariate analysis between various considered parameters and incident DM. The univariate analysis results demonstrated that age, ethanol consumption, smoking status, SBP, DBP, BMI, WC, fatty liver, TG, TC, HbA1c, and FPG were positively related to the incident diabetes. It showed that low HDL-C levels were associated with increased risk for diabetes in univariate analysis. Compared with female participants, male participants were more likely to develop DM.We stratified the Kaplan–Meier curves of the cumulative hazards of diabetes incident risk by HDL-C quartiles in Fig. 1. The diabetes incident risk was significantly different among the four HDL-C groups (*p* < 0.0001). The cumulative diabetes incident risk gradually increased with decreased HDL-C. Therefore, the lowest HDL-C group has the greatest risk of diabetic events.

**Table 2:** The results of univariate analysis

	Statistics	HR (95% CI)	*P* value
Gender			
Women	6941 (45.25%)	ref	
Men	8397 (54.75%)	2.51 (1.98, 3.20)	< 0.0001
Age(years)	43.69 ± 8.89	1.06 (1.04, 1.07)	< 0.0001
Ethanol consumption(g/week)	47.67 ± 82.24	1.00 (1.00, 1.00)	0.0011
Smoking status			
Never-smoker	8940 (58.29%)	ref	
Ex-smoker	2928 (19.09%)	1.65 (1.25, 2.18)	0.0004
Current-smoker	3470 (22.62%)	2.58 (2.05, 3.24)	< 0.0001
Regular exerciser			
No	12,657 (82.52%)	ref	
Yes	2681 (17.48%)	0.76 (0.57, 1.02)	0.0686
SBP (mmHg)	114.51 ± 14.97	1.03 (1.03, 1.04)	< 0.0001
DBP (mmHg)	71.60 ± 10.50	1.05 (1.04, 1.06)	< 0.0001
BMI (kg/m^2^)	22.13 ± 3.13	1.24 (1.22, 1.27)	< 0.0001
WC (cm)	76.52 ± 9.10	1.09 (1.08, 1.10)	< 0.0001
Fatty liver			
No	12,604 (82.17%)	ref	
Yes	2734 (17.83%)	6.99 (5.69, 8.60)	< 0.0001
ALT (IU/L)	20.00 ± 14.38	1.01 (1.01, 1.01)	< 0.0001
AST (IU/L)	18.39 ± 8.59	1.01 (1.01, 1.01)	< 0.0001
HDL-C (mmol/L)	1.45 ± 0.38	0.13 (0.09, 0.18)	< 0.0001
TG (mmol/L)	0.92 ± 0.66	1.80 (1.68, 1.92)	< 0.0001
TC (mmol/L)	5.12 ± 0.86	1.50 (1.35, 1.67)	< 0.0001
HbA1c (%)	5.17 ± 0.32	55.87 (40.56, 76.97)	< 0.0001
FPG (mmol/L)	5.16 ± 0.41	25.46 (18.77, 34.55)	< 0.0001

### The multivariate analysis of HDL-C with DM risk

In Table 3, multivariate analysis was applied to assess the relationships between HDL-C and DM risk, including crude and adjusted models. Low HDL-C levels were associated with increased risk for diabetes in the unadjusted model (HR = 0.13, 95% CI: 0.09 to 0.18, *P* < 0.0001). In model I (adjusted gender, age, ethanol consumption, smoking status, regular exerciser, SBP, DBP, BMI, WC), the result still existed (HR: 0.34, 95% CI: 0.23 to 0.50). After adjusting for gender, age, ethanol consumption, smoking status, regular exerciser, SBP, DBP, BMI, WC, TC, TG, HbA1c, FPG in the model II, the connection could also be detected (HR = 0.54, 95%CI: 0.35 to 0.82, *P* = 0.0044). When converting HDL-C into a categorical variable, the relationship between HDL-C and diabetes in the highest quartile is not statistically significant compared to the lowest quartile in model II (HR = 0.67, 95%CI: 0.43 to 1.05, *P* = 0.0791). However, the trend across the quartiles was significant in model II (*P* = 0.0056). The *p* trend became non-equidistant when HDL-C was converted to a categorical variable, indicating a non-linear relationship between HDL-C and the DM risk (Table 3). Then GAM was performed to insert the continuity covariate into the equation as a curve. It generally remained consistent with the GAM (HR: 0.47; 95% CI:0.30 to 0.75, *P* = 0.0015), which demonstrated the robustness of the results (Table 3). Then we stratified the adjusted model by gender, for every unit increased in HDL-C ratio, the incidence of diabetes decreased by 55% in males (HR = 0.45; 95%CI: 0.27–0.76) and 8% in females (HR = 0.92; 95%CI: 0.41–2.07). Besides, we generated an E-value to assess the sensitivity to unmeasured confounding. The E-value was 3.11. The E-value was greater than the relative risk of unmeasured confounders and HDL-C, suggesting unmeasured or unknown confounders had little effect on the relationship between HDL-C and incident diabetes.

**Table 3:** Relationship between HDL-C and the incident diabetes in different models

Variable	Crude model (HR.,95% CI, P)	Model I (HR,95% CI, P)	Model II (HR,95% CI, P)	GAM (HR,95% CI, P)
**Total**				
HDL-C	0.13 (0.09, 0.18) < 0.0001	0.34 (0.23, 0.50) < 0.0001	0.54 (0.35, 0.82) 0.0044	0.47 (0.30, 0.75) 0.0015
HDL-C(quartile)				
Q1	ref	ref	ref	ref
Q2	0.53 (0.42, 0.68) < 0.0001	0.68 (0.53, 0.87) 0.0023	0.83 (0.64, 1.07) 0.1575	0.82 (0.63, 1.06) 0.1343
Q3	0.28 (0.20, 0.39) < 0.0001	0.44 (0.31, 0.63) < 0.0001	0.58 (0.41, 0.83) 0.0031	0.54 (0.37, 0.79) 0.0014
Q4	0.23 (0.15, 0.34) < 0.0001	0.45 (0.30, 0.70) 0.0003	0.67 (0.43, 1.05) 0.0791	0.58 (0.36, 0.94) 0.0266
*P* for trend	< 0.0001	< 0.0001	0.0056	0.0018
**Female**				
HDL-C	0.14 (0.07, 0.28) < 0.0001	0.41 (0.20, 0.84) 0.0155	0.92 (0.41, 2.07) 0.8386	1.04 (0.44, 2.44) 0.9330
HDL-C(quartile)				
Q1	ref	ref	ref	ref
Q2	0.47 (0.27, 0.81) 0.0070	0.67 (0.39, 1.17) 0.1606	1.07 (0.58, 1.98) 0.8178	1.13 (0.61, 2.06) 0.7021
Q3	0.21 (0.11, 0.38) < 0.0001	0.41 (0.22, 0.77) 0.0056	0.73 (0.36, 1.49) 0.3869	0.77 (0.38, 1.55) 0.4589
Q4	0.17 (0.09, 0.32) < 0.0001	0.43 (0.22, 0.85) 0.0145	0.90 (0.41, 1.99) 0.8013	1.01 (0.45, 2.28) 0.9841
*P* for trend	< 0.0001	0.0051	0.5208	0.6933
**Male**				
HDL-C	0.17 (0.11, 0.26) < 0.0001	0.32 (0.20, 0.51) < 0.0001	0.45 (0.27, 0.76) 0.0030	0.37 (0.21, 0.65) 0.0005
HDL-C(quartile)				
Q1	ref	ref	ref	ref
Q2	0.53 (0.40, 0.70) < 0.0001	0.66 (0.50, 0.87) 0.0035	0.79 (0.59, 1.06) 0.1097	0.76 (0.56, 1.02) 0.0713
Q3	0.31 (0.20, 0.46) < 0.0001	0.46 (0.30, 0.70) 0.0003	0.57 (0.37, 0.89) 0.0131	0.51 (0.32, 0.82) 0.0052
Q4	0.28 (0.16, 0.50) < 0.0001	0.50 (0.28, 0.92) 0.0248	0.63 (0.34, 1.17) 0.1411	0.45 (0.23, 0.88) 0.0200
*P* for trend	< 0.0001	< 0.0001	0.0080	0.0011

Crude model: we did not adjust for other covariants

Model I: we adjusted for age, ethanol consumption, smoking, regular exerciser, SBP, DBP, BMI, WC

Model II: we adjusted for age, ethanol consumption, smoking, regular exerciser, SBP, DBP, BMI, WC, TC, TG, HbA1c, FPG

GAM: All covariates listed in Table 1 were adjusted. However, continuous covariates were adjusted as nonlinearity

*CI* Confidence interval, *Ref* Reference

### The analyses of non-linear relationship between HDL-C and DM risk

**Fig. 2:**
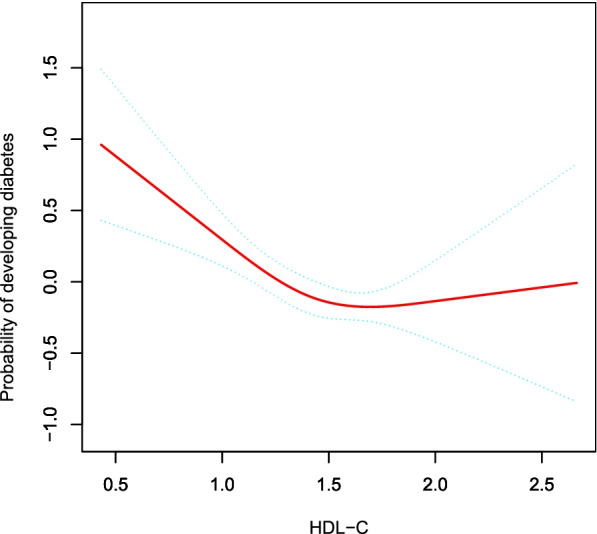
The non-linear relationship between HDL-C and incident diabetes. A non-linear relationship between them was detected after adjusting for gender, age, ethanol consumption, smoking, regular exerciser, SBP, DBP, BMI, WC, TC, TG, HbA1c, FPG

GAM was applied to assess whether there was a non-linear relationship between HDL-C and DM risk in our study (Fig. 2). A non-linear relationship between HDL-C and DM risk was discovered after adjusting for gender, age, ethanol consumption, smoking status, regular exerciser, SBP, DBP, BMI, WC, TC, TG, HbA1c and FPG (Log-likelihood ratio test *P* = 0.005). The inflection point of HDL-C was detected to be 1.72 mmol/L by employing a two-piecewise linear regression model. When HDL-C ≤ 1.72 mmol/L, we observed that low HDL-C levels were associated with increased risk for diabetes (HR: 0.36, 95%CI: 0.21 to 0.59, *P* < 0.0001). However, when HDL-C > 1.72 mmol/L, the result showed that elevated HDL-C levels might be associated with an increased risk of diabetes, but the results were not statistically significant(HR: 2.90, 95%CI: 0.96 to 8.75, *P* = 0.0594) (Table 4).

**Table 4:** The result of two-piecewise linear regression model

	Incident diabetes	(HR,95%CI, P)
Fitting model by standard linear regression	0.54 (0.35, 0.82)	0.0044
Fitting model by two-piecewise linear regression		
Inflection point of HDL-C	1.72	
≤ 1.72	0.36 (0.21, 0.59)	< 0.0001
> 1.72	2.90 (0.96, 8.75)	0.0594
P for log likelihood ratio test	0.005	

We adjusted for gender, age, ethanol consumption, smoking, regular exerciser, SBP, DBP, BMI, WC, TC, TG, HbA1c, FPG

*CI* Confidence interval

### The results of subgroup analyses

Subgroup analysis was applied to discover potential confounding variables that might affect the association between HDL-C and DM risk. As shown in Table 5, we chose age, gender, ethanol consumption, smoking status, regular exerciser, SBP, DBP, BMI, WC, fatty liver as the stratification variables and further detected the trend of effect sizes in these stratification variables. Based on our a priori specification, we discovered most stratification variables did not affect the relationship between HDL-C and DM risk, except smoking status, SBP and DBP (*P* < 0.05). A stronger association was observed in ex-smokers, current-smokers and subjects with arterial hypertension (SBP ≥ 140 mmHg or DBP ≥ 90 mmHg) in our study. In contrast, there was a weaker association in the people with never-smoker, SBP < 140 mmHg, and DBP < 90 mmHg.

**Table 5:** Effect size of HDL-C on diabetes in prespecified and exploratory subgroups

Characteristic	No of patients	Effect size(95%CI)	*P* value	*P* for interaction
Age(years)				0.3924
< 60	14,633	0.64 (0.41, 1.01)	0.0543	
≥ 60	705	0.33 (0.07, 1.49)	0.1497	
Gender				0.2075
Women	6941	0.84 (0.39, 1.81)	0.6515	
Men	8397	0.46 (0.27, 0.78)	0.0036	
Ethanol consumption (g/week)				0.4092
0	11,718	0.70 (0.43, 1.14)	0.1508	
> 0	3620	0.51 (0.27, 0.98)	0.0442	
Smoking status				0.0062
Never-smoker	8940	1.28 (0.68, 2.42)	0.4420	
Ex-smoker	2928	0.29 (0.10, 0.83)	0.0212	
Current-smoker	3470	0.32 (0.16, 0.66)	0.0020	
Regular exerciser				0.9793
No	12,657	0.54 (0.34, 0.84)	0.0070	
Yes	2681	0.54 (0.22, 1.34)	0.1860	
SBP (mmHg)				0.0160
< 140	14,556	0.76 (0.48, 1.19)	0.2275	
≥ 140	782	0.14 (0.03, 0.56)	0.0055	
DBP (mmHg)				0.0055
< 90	14,578	0.78 (0.50, 1.22)	0.2694	
≥ 90	760	0.09 (0.02, 0.42)	0.0023	
BMI (kg/m^2^)				0.2004
< 25	12,817	0.71 (0.42, 1.20)	0.1952	
≥ 25	2521	0.40 (0.19, 0.82)	0.0131	
Visceral fat obesity				0.9829
No	13,332	0.60 (0.37, 0.98)	0.0399	
Yes	2006	0.59 (0.29, 1.23)	0.1618	
Fatty liver				0.7475
No	12,604	0.67 (0.39, 1.16)	0.1561	
Yes	2734	0.59 (0.32, 1.11)	0.1025	

Note 1: Above model adjusted for gender, age, ethanol consumption, smoking, regular exerciser, SBP, DBP, BMI, WC, TC, TG, HbA1c, FPG

Note 2: In each case, the model is not adjusted for the stratification variable

## Discussion

In this retrospective cohort study, we found that low HDL-C was independently related to DM risk after adjusting for gender, age, ethanol consumption, smoking status, regular exerciser, SBP, DBP, BMI, WC, TC, TG, HbA1c, FPG. Further analysis showed a non-linear relationship between HDL-C level and DM risk (Log-likelihood ratio test *P* = 0.005). This result suggested that low HDL-C levels were associated with increased risk for diabetes when the HDL-C level was ≤ 1.72 mmol/L (HR: 0.36, 95%CI: 0.21 to 0.59, *P* < 0.0001). However, when HDL-C > 1.72 mmol/L, the result showed that elevated HDL-C levels might be associated with an increased risk of diabetes, but the results were not statistically significant (HR: 2.90, 95%CI: 0.96 to 8.75, *P* = 0.0594). It could be better understood the trend of HDL-C and diabetes incidence in different populations by analysis of sub-groups. A stronger association between HDL-C and DM risk was discovered in the participants with ex-smoker, current-smoker and hypertension (SBP ≥ 140 mmHg or DBP ≥ 90 mmHg). In contrast, there was a weaker association in never-smokers and subjects with SBP < 140 mmHg.

Cardiovascular disease is a significant cause of morbidity and mortality in type 2 diabetes mellitus patients [22], and HDL-C plays an essential role in cardiovascular disease and diabetes. It was reported that low mean and high variability of HDL-C were independent predictors of diabetes with an additive effect [23]. Our study found that low HDL-C is an independent risk of DM, which was consistent with previous studies [24]. In recent prospective research with a multivariable-adjusted logistic regression analysis model, Abbasi et al. found that HDL-C was negatively associated with the risk of DM in 6820 patients without diabetes (OR = 0.74, 95%CI: 0.61–0.88, *P* = 0.001) after adjustment for age, sex, family history of diabetes, BMI, hypertension, alcohol, smoking, FPG and TG [8]. In a retrospective cohort study that included 5947 Caucasian participants aged 28–75 years with no pre-existing diabetes, Kunutsor et al. found that baseline HDL-C levels were also negatively associated with DM through multivariate Cox proportional hazards model analysis(HR = 0.79, 95%CI: 0.68–0.92, *P* = 0.002) after adjusting age, sex, FPG, BMI, SBP, smoking status, alcohol consumption, parental history of diabetes, TG, estimated glomerular filtration rate, loge urinary albumin excretion, and loge homeostasis model assessment of insulin resistance, paraoxonase-1 [9]. Our study found similar findings to some of the previous studies, but our study had a large sample size and excluded participants with FPG ≥ 6.1 mmol/L compared to previous studies. In addition, we further validated the association between HDL-C and diabetes risk by using sensitivity analysis, applying subgroup analysis, using a GAM to insert the continuity covariate into the equation as a curve, applying generalized additive model and a smooth curve fitting to explore the non-linear relationship, calculating E-values, and adjusting for more confounding variables (WC, regular exerciser). The efforts mentioned above have confirmed the relationship’s stability between HDL-C and DM risk. The results provided a reference for clinical intervention in HDL-C levels to reduce the risk of DM. Our findings extend the existing literature that supports the hypothesis that reduced HDL-C increases the risk of developing diabetes.

HDL-C affected the incident diabetes through different mechanisms. Previous studies have also revealed that HDL also has anti-inflammatory and antioxidant activities [25]. HDL can also directly enhance the survival of islet β cell by inhibiting apoptosis due to oxidised low-density lipoprotein or inflammatory cytokines, such as tumour necrosis factoralpha. The anti-apoptotic response may be related to reduced activation of the endoplasmic reticulum stress response, as well as inhibition of intracellular reactive oxygen species, thereby, preventing cytochrome c release from mitochondria and activation of the caspase cascade [26]. Besides, HDL-C improves cell sensitivity to insulin and glucose uptake in skeletal muscle cells by activating Adenosine 5’-monophosphate (AMP)-activated protein kinase (AMPK) pathway [27]. Moreover, HDL-C can promote insulin secretion by increasing the outflow of cholesterol from pancreatic β cells [28]. These mechanisms can provide a reasonable explanation for the association between the reduction of HDL-C and the increase of the risk of DM.

In the past, it was generally believed that higher HDL-C was more beneficial. However, Lin et al. [29] found that high serum HDL-C levels increase the risk of DM after adjusting for the demographic and clinical covariates in a retrospective study of 9764 Chinese. A recent multicenter retrospective study involving 114,787 participants showed that high levels of HDL-C increase the risk of DM [10]. Also, Chen et al. [13] found high serum HDL-C levels increase the risk of DM after adjusting for potential confounders in a large retrospective cohort study of 3414 patients. A population-based study of 328 patients in 2016 showed that patients with scavenger receptor BIP376L gene mutations have significantly increased plasma HDL-C levels, but the risk of coronary heart disease is also increased, but the risk of coronary heart disease was increased [30]. Animal studies showed that 8 SR-BI knockout mice increased HDL-C levels considerably, but the probability of atherosclerosis also increased [31]. Compared to these studies mentioned above, the reason for the inconsistent results might come from the following: (1) The study population was different. These studies that were inconsistent with our results mainly focused on China, Canada, and the United States. (2) Many studies with those different conclusions did not clearly clarify the nonlinear relationship and used different regression models. (3) Compared with our research, those studies did not consider the effect of regular exerciser, WC on the association between HDL-C and incident diabetes when adjusting covariates. However, previous studies considered these variables as factors related to HDL-C or diabetes risk. (4) This might be related to the different exclusion criteria. Participants with prior drug use, heavy drinking, and baseline FPG ≥ 6.1 mmol/L were excluded from our study. (5) This might also be related to different HDL-C levels. Furthermore, to the best of our knowledge, the present study observed a non-linear relationship between HDL-C and diabetes risk for the first time. The current study used a two-piecewise Cox proportional hazards regression model to clarify a non-linear relation between HDL-C and diabetes risk. The inflection point was 1.72 mmol/L after adjusting for confounders. It showed that when HDL-C was below 1.72 mmol/L, a 1 unit decrease in the HDL-C level was associated with a 64% greater adjusted HR of diabetes risk (HR = 0.36, 95%CI: 0.21–0.59). However, when HDL-C > 1.72 mmol/L, a 1 unit increase in HDL-C level was associated with 1.9 times greater adjusted HR of the risk of diabetes (HR = 2.90, 95%CI: 0.96–8.75), but the results were not statistically significant. The reason is that other variables in the participants’ baseline may also have influenced DM risk. In addition, the excessive elevation of HDL-C may increase the risk of atherosclerosis [31] which plays an important role in the development of diabetes [32]. Therefore, it is possible that higher HDL-C levels may increase the risk of diabetes. Our findings provide an essential rationale for preventing diabetes by intervening in the HDL-C level in the clinic. The inflection point provides evidence for the first time for HDL-C management in the Japanese population. Therefore, this assay has excellent clinical value. Thus, this study can expand the current research on the relationship between HDL-C and diabetes risk.

The current study has several strengths. (1) We used a GAM model and a smooth curve fitting (penalty curve method) to explore the non-linear relationship; therefore, our analysis has greater clinical value, which has not been explored in previous studies. (2) Strict statistical adjustments were used to minimize residual confounding factors. (3) In this study, we ensured the robustness of the results through a series of sensitivity analyses (conversion of target-independent variable form, subgroup analysis, using a GAM to insert the continuity covariate into the equation as a curve, calculating E-values to explore the potential for unmeasured confounding).

The current study has several following limitations. Firstly, the retrospective cohort study was based on the personal medical records of Murakami Memorial Hospital in Japan, and Takuro Okamura et al. screened the data. We could not conclude that our conclusion could be generalized to other races, areas, and some unique populations because the participants were all from Japan. We have conducted subgroup analysis by age, gender, etc., and found that the relationship between HDL-C and diabetes risk remains stable in different age and gender populations. In the future, we could also consider designing our study or collaborating with other researchers to explore the relationship between HDL-C and diabetes risk in different people and ethnicities. Secondly, the incidence of diabetes may be underestimated because the original research lacked a 2 h oral glucose tolerance test to diagnose DM. However, it was not feasible to perform 2 h oral glucose tolerance tests for all participants due to a lack of financial and logistical support. In the future, we can consider designing our studies and performing a 2 h oral glucose tolerance test to diagnose diabetes more accurately. Thirdly, only baseline HDL-C was measured in the previous original study, and the original research did not involve changes in HDL-C over time. In the future, we can consider designing our studies to collect as many variables as possible, including information on the evolution of HDL-C during follow-up. Therefore, we can observe the changes in HDL-C before and after treatment and further explore the effect of changes in HDL-C on the risk of diabetes in the future through a generalized additive mixed model. Finally, our research could not exclude some residual or unmeasured confounding factors, such as dietary factors and family history of diabetes, which may bias the results. However, the authors calculated the E-value to quantify the potential impact of unmeasured confounders and found that unmeasured confounders were unlikely to explain the results.

## Conclusion

This study demonstrates a negative and non-linear relationship between HDL-C and diabetes in the Japanese population. There is a threshold effect between HDL-C and diabetes. When HDL-C is lower than 1.72 mmol/L, the decreased HDL-C levels were associated with an increased risk for diabetes. This study provides a reference for the future risk of diabetes in people with different HDL-C levels.

## Supplementary Information

**Additional file 1: Table 1S.** The Baseline Characteristics of participants.

## Data Availability

The data are available from the ‘DataDryad’ database (https://datadryad.org/stash/dataset/doi:10.5061%2Fdryad.8q0p192).

## Abbreviations

BMI: Body mass index
WC: Waist circumference
CVD: Cardiovascular diseases
SBP: Systolic blood pressures
DBP: Diastolic blood pressures
ALT: Alanine aminotransferase
AST: Aspartate aminotransferase
HDL-C: High-density lipoprotein cholesterol
TC: Total cholesterol
TG: Triglycerides
HbA1c: Hemoglobin A1c
FPG: Fasting plasma glucose
GAM: Generalized additive models
CETP: Cholesteryl ester transfer protein
DM: Diabetes mellitus
HR: Hazard ratio
SD: Standard deviations
CI: Confidence interval
