# Supplementary material for: Non-linear relationship between high-density lipoprotein cholesterol and incident diabetes mellitus: a secondary retrospective analysis based on a Japanese cohort study
Source: BMC Endocr Disord. 2022 Jun 18;22:163. doi: 10.1186/s12902-022-01074-8 (PMC9206738; doi:10.1186/s12902-022-01074-8)
Supplement: Supplementary file 1 — Additional file 1: Table 1S. The Baseline Characteristics of participants. [file 12902_2022_1074_MOESM1_ESM.docx]

**Table 1S** The Baseline Characteristics of participants

| HDL-C(mmol/L) | Q1(≤1.16) | Q2(1.16 to ≤1.41) | Q3(1.41 to ≤1.70) | Q4(>1.70) | P (Q1 VS. Q2) | P (Q1 VS. Q3) | P (Q1 VS. Q4) | P (Q2 VS. Q3) | P (Q2 VS. Q4) | P (Q3 VS. Q4) | P |
| --- | --- | --- | --- | --- | --- | --- | --- | --- | --- | --- | --- |
| Participants | 3752 | 3895 | 3845 | 3846 |  |  |  |  |  |  |  |
| Gender |  |  |  |  | <0.001 | <0.001 | <0.001 | <0.001 | <0.001 | <0.001 | <0.001 |
| Women | 568 (15.14%) | 1419 (36.43%)^a^ | 2122 (55.19%)^ab^ | 2832 (73.63%)^abc^ |  |  |  |  |  |  |  |
| Men | 3184 (84.86%) | 2476 (63.57%) | 1723 (44.81%) | 1014 (26.37%) |  |  |  |  |  |  |  |
| Age(years) | 44.49 ± 9.03 | 43.63 ± 8.96^a^ | 43.31 ± 8.77^a^ | 43.36 ± 8.78^a^ | <0.001 | <0.001 | <0.001 | 0.107 | 0.184 | 0.780 | <0.001 |
| Ethanol consumption(g/week) | 4.20(0, 66.00) | 1.00(0, 72.00) | 1.00(0, 60.00) | 1.00(0, 54.00)^abc^ | 0.700 | 0.156 | <0.001 | 0.069 | <0.001 | 0.002 | <0.001 |
| Smoking status |  |  |  |  | <0.001 | <0.001 | <0.001 | <0.001 | <0.001 | <0.001 | <0.001 |
| Never-smoker | 1436 (38.27%) | 2114 (54.27%)^a^ | 2484 (64.60%)^ab^ | 2906 (75.56%)^abc^ |  |  |  |  |  |  |  |
| Ex-smoker | 863 (23.00%) | 821 (21.08%) | 694 (18.05%) | 550 (14.30%) |  |  |  |  |  |  |  |
| Current-smoker | 1453 (38.73%) | 960 (24.65%) | 667 (17.35%) | 390 (10.14%) |  |  |  |  |  |  |  |
| Regular exerciser |  |  |  |  | 0.009 | <0.001 | <0.001 | 0.390 | 0.003 | 0.036 | <0.001 |
| No | 3190 (85.02%) | 3226 (82.82%)^a^ | 3156 (82.08%)^a^ | 3085 (80.21%)^abc^ |  |  |  |  |  |  |  |
| Yes | 562 (14.98%) | 669 (17.18%) | 689 (17.92%) | 761 (19.79%) |  |  |  |  |  |  |  |
| SBP (mmHg) | 118.88 ± 14.75 | 115.96 ± 14.97^a^ | 112.67 ± 14.53^ab^ | 110.63 ± 14.31^abc^ | <0.001 | <0.001 | <0.001 | <0.001 | <0.001 | <0.001 | <0.001 |
| DBP (mmHg) | 75.00 ± 10.21 | 72.62 ± 10.41^a^ | 70.29 ± 10.23^ab^ | 68.55 ± 10.03^abc^ | <0.001 | <0.001 | <0.001 | <0.001 | <0.001 | <0.001 | <0.001 |
| BMI (kg/m^2^) | 23.95 ± 3.11 | 22.61 ± 3.08^a^ | 21.50 ± 2.71^ab^ | 20.52 ± 2.47^abc^ | <0.001 | <0.001 | <0.001 | <0.001 | <0.001 | <0.001 | <0.001 |
| WC (cm) | 82.37 ± 8.30 | 78.17 ± 8.52^a^ | 74.47 ± 8.13^ab^ | 71.20 ± 7.40^abc^ | <0.001 | <0.001 | <0.001 | <0.001 | <0.001 | <0.001 | <0.001 |
| Fatty liver |  |  |  |  | <0.001 | <0.001 | <0.001 | <0.001 | <0.001 | <0.001 | <0.001 |
| No | 2339 (62.34%) | 3081 (79.10%)^a^ | 3491 (90.79%)^ab^ | 3693 (96.02%)^abc^ |  |  |  |  |  |  |  |
| Yes | 1413 (37.66%) | 814 (20.90%) | 354 (9.21%) | 153 (3.98%) |  |  |  |  |  |  |  |
| ALT (IU/L) | 21(16.00,30.00) | 17 (13.00,24.00)^a^ | 15 (12.00,20.00)^ab^ | 14(11.00,19.00)^abc^ | <0.001 | <0.001 | <0.001 | <0.001 | <0.001 | <0.001 | <0.001 |
| AST (IU/L) | 18 (15.00,22.00) | 17 (14.00,21.00)^a^ | 17 (14.00,20.00)^ab^ | 17 (13.00,20.00)^abc^ | <0.001 | <0.001 | <0.001 | <0.001 | <0.001 | 0.035 | <0.001 |
| HDL-C (mmol/L) | 1.00 ± 0.12 | 1.29 ± 0.07^a^ | 1.54 ± 0.08^ab^ | 1.97 ± 0.22^abc^ | <0.001 | <0.001 | <0.001 | <0.001 | <0.001 | <0.001 | <0.001 |
| TG (mmol/L) | 1.20 (0.83,1.73) | 0.82 (0.56,1.17)^a^ | 0.64 (0.45,0.88)^ab^ | 0.53 (0.38,0.72)^abc^ | <0.001 | <0.001 | <0.001 | <0.001 | <0.001 | <0.001 | <0.001 |
| TC (mmol/L) | 5.07 ± 0.89 | 5.07 ± 0.89 | 5.07 ± 0.84 | 5.27 ± 0.80^abc^ | 0.972 | 0.784 | <0.001 | 0.809 | <0.001 | <0.001 | <0.001 |
| HbA1c (%) | 5.19 ± 0.34 | 5.18 ± 0.33 | 5.15 ± 0.31^ab^ | 5.17 ± 0.30^ac^ | 0.077 | <0.001 | 0.024 | <0.001 | 0.616 | <0.001 | <0.001 |
| FPG (mmol/L) | 5.30 ± 0.38 | 5.20 ± 0.40^a^ | 5.11 ± 0.41^ab^ | 5.04 ± 0.41^abc^ | <0.001 | <0.001 | <0.001 | <0.001 | <0.001 | <0.001 | <0.001 |

Values are n (%) or mean ± SD

SBP systolic blood pressures, DBP diastolic blood pressures, BMI body mass index, WC waist circumference, ALT alanine aminotransferase, AST aspartate aminotransferase, HDL-C high-density lipoprotein cholesterol, TC total cholesterol, TG triglycerides, HbA1c hemoglobin A1c, FPG fasting plasma glucose

^a^ represents other groups (Q2, Q3, Q4) compared with Q1, P<0.05.

^b^ represents other groups (Q3, Q4) compared with Q2, P<0.05.

^c^ represents other groups (Q4) compared with Q3, P<0.05.
